# Venous thromboembolic disease in admitted blunt trauma patients: what matters?

**DOI:** 10.1186/s12959-023-00555-7

**Published:** 2023-10-27

**Authors:** Camille Lineberry, Dimitri Alexis, Ambika Mukhi, Kevin Duh, Mathew Tharakan, James A. Vosswinkel, Randeep S. Jawa

**Affiliations:** 1 Division of Trauma, Department of Surgery, Stony Brook University Renaissance School of Medicine, Stony Brook, NY USA; 2 Department of Medicine, Stony Brook University Renaissance School of Medicine, Stony Brook, NY USA

**Keywords:** Trauma, Venous thromboembolism, Deep venous Thrombosis, Pulmonary Embolism

## Abstract

**Background:**

Venous thromboembolic events (VTE) are a significant cause of morbidity and mortality following traumatic injury. We examined demographic characteristics, chemoprophylaxis, and outcomes of VTE patients with blunt trauma requiring hospitalization.

**Methods:**

A retrospective review of adult blunt trauma hospitalizations with and without VTE between 2012 and 2019 was conducted. Deaths in the emergency department were excluded. Univariate and multivariable analyses, including machine learning classification algorithms for VTE, were performed.

**Results:**

Of 10,926 admitted adult blunt trauma patients, 177 had VTE events. VTE events occurred at a median of 6 [IQR 3–11] days, with 7.3% occurring within 1 day of admission. VTE patients were more often male, and more often underwent surgery. They had higher injury severity as well as longer intensive care unit and hospital lengths of stay. While VTE occurred throughout the spectrum of injury severity, 27.7% had low injury severity (ISS < = 9). In multivariable analyses, both heparin and enoxaparin had reduced adjusted odds ratios for VTE.

**Conclusion:**

Approximately 7.3% of VTE events occurred within one day of admission. A substantial proportion of VTE events occurred in patients with low injury severity (ISS < = 9). Subcutaneous unfractionated heparin and enoxaparin chemoprophylaxis were both inversely associated with VTE. These findings underscore the need for vigilance for VTE identification in blunt trauma patients throughout their hospitalization and VTE prevention efforts.

## Introduction

Venous thromboembolism (VTE) events, including deep vein thrombosis (DVT) and pulmonary embolism (PE), continue to be a major cause for morbidity and mortality for hospitalized trauma patients. Albeit VTE events are commonly reported as having a higher frequency in trauma patients than in the general population, a very wide range in VTE incidence of 1–63% has been reported. This may partly be based on VTE prophylaxis and surveillance strategies [1–10]. Primary prevention methods for VTE include early mobilization, lower extremity pneumatic compression devices, and chemoprophylaxis, although for multiple reasons, there is protocol variability at trauma centers [2, 11–13]. Recent guidelines published by the American Association for the Surgery of Trauma (AAST) and the Western Trauma Association (WTA) address this issue by emphasizing the importance of identifying and targeting at-risk subgroups, while acknowledging that chemoprophylaxis decision-making is complex [2, 6, 14]. Given the variability in reported VTE incidence rates, we examined demographic characteristics, chemoprophylaxis agents, and outcomes of VTE patients to further define the at-risk population.

## Methods

The trauma registry at our regional/Level 1 trauma center was retrospectively queried for all hospitalized adult blunt trauma patients discharged between April 2012 to August 2019. The registry is prospectively maintained by trained trauma nurse registrars. Comorbidities and complications are encoded according to the National Trauma Data Standard (NTDS). Study exclusion criteria included death in the emergency department, age < 18 years, or penetrating mechanism of injury. A retrospective review of the medical records of patients with VTE was subsequently conducted. This study was approved by our Institutional Review Board.

Diagnosis of extremity DVT was based on duplex ultrasonography. Of note, duplex ultrasonography was not protocolized during the study period. However, common reasons for its utilization included symptoms/clinical findings, contraindications to or pauses in VTE prophylaxis, or as serial surveillance imaging in patients with infrageniculate DVT who were not treated with therapeutic anticoagulation. Any lower extremity thrombosis involving proximal lower extremity veins (i.e. popliteal to external iliac vein), regardless of subsequent management strategy, was classified as a DVT. Infrageniculate thromboses were counted as a DVT only if the thrombus was treated with therapeutic anticoagulation or inferior vena cava (IVC) filter placement, in accordance with Trauma Quality Improvement Project (TQIP) guidelines. Upper extremity thromboses were classified as DVT if they involved the brachial, axillary, or subclavian veins, or any combination thereof, regardless of management strategy.

Diagnosis of PE was largely based on CT chest with IV contrast for clinical suspicion of PE. However, many patients received CT chest imaging as part of their initial trauma evaluation. Ventilation/perfusion scanning was infrequently performed. Patients may also have had combinations of DVT and PE and/or DVT in more than one location on the same or different day.

Chemoprophylaxis is generally ordered on adult trauma patients at admission with exceptions for those at high risk for bleeding complications. The choice of agent during the study period was at the discretion of the attending physician, however, heparin was generally preferentially used in patients with renal impairment, patients who may require epidural analgesia/anesthesia, pre-operative patients, and those at high-risk for bleeding complications. By convention, prophylactic subcutaneous unfractionated heparin dosing was generally 5,000 units every 8 h. Prophylactic enoxaparin dosing was generally 40 mg subcutaneous daily; 30 mg twice daily was infrequently prescribed. Of note, these dosing regimens preceded the recent WTA and AAST guidelines which generally recommend higher doses of enoxaparin for trauma patients [2, 6]. Earlier during the study period, patients with injuries were admitted to a variety of medical and surgical services. With the transition to American College of Surgeons level I trauma center verification, patients were transitioned to trauma center admission with concomitant decrease in practice variation. Mechanical VTE prophylaxis has been and continues to be routinely requested for all trauma admissions.

Demographics, risk factors for VTE development, and outcomes were examined via univariate statistics including Chi Square, Fisher exact, Wilcoxon rank-sum, and Kruskal-Wallis tests, as appropriate, using R software for statistical computing (Vienna, Austria). Multivariable models were also developed to evaluate risk factors. Risk factors examined included age; sex; mechanism of injury (motor vehicle crash/motorcycle crash, fall, other); number of comorbidities; intensive care unit (ICU) stay; mechanical ventilation; any surgery (excluding IVC filters) done in the operating room; total number of non-VTE complications; presence and type of chemical VTE prophylaxis (none, heparin, enoxaparin); and the Abbreviated Injury Score (AIS) in each body region (head/neck, chest, abdomen, extremity, external, and face).

With regards to chemoprophylaxis characterization in patients with VTE, if a patient received an initial dose of chemical DVT prophylaxis on the same day as the VTE event, prophylaxis was counted as none. Chemoprophylaxis was counted as present as long as at least one dose was administered on the day prior to the event. Patients who were sequentially on heparin and enoxaparin prior to the VTE event were classified as being on heparin in these analyses as that was the index agent in all VTE cases and thereby facilitated comparison with the index chemoprophylaxis agent in the no VTE group, where changing of agent may also have occurred. Patients on other chemoprophylactic agents were excluded in multivariable models because of the small numbers.

The AIS measures injury severity in six body regions. A score of 0 indicates no injury, 3 a severe injury, and a maximal score of 6 indicates a generally non-survivable injury. The maximal score varies by body region, e.g., head/neck injury has a maximal value of 6. The ISS score, a measure of global injury burden, ranges from 0 to 75, and is calculated by squaring the AIS of the 3 most severely injured body regions and adding these values together. A score of 0 indicates no injury. A score of 75 indicates an often unsurvivable injury; concomitantly, an AIS of 6 is automatically given an ISS of 75. As AIS in each body region was considered in multivariable models, ISS was excluded because of collinearity concerns.

Because of the highly imbalanced dataset with few VTE patients, several different multivariable classification algorithms based on an 80:20 training/test dataset were examined, including logistic regression, random forest, and gradient boosting machines (GBM). Random forest and GBM models are both machine learning ensemble decision-tree based models. Whereas random forest builds trees independent of previous trees, GBM models sequentially build trees to address errors made by previous trees. The hyperparameter tuned for random forest was the number of variables considered at each split of a tree. For GBM, hyperparameters tuned included the minimum numbers of observations per node, shrinkage, interaction depth, and number of trees. Cross-validation was used to facilitate model tuning.

To further address class imbalance, the Youden index (threshold at which sensitivity + specificity − 1 is maximal) was utilized to determine the probabilistic odds threshold for classification in logistic regression and GBM models with the raw data. In addition, synthetic data was generated from the scaled training dataset, whereby the minority class (VTE patients) were oversampled while the majority class (no VTE patients) were undersampled, to create a fully balanced training dataset. This synthetic dataset was then utilized for generating new models that were tested on the scaled testing dataset.

To better evaluate model performance with this imbalanced dataset, balanced accuracy (sensitivity and specificity/2) and F1 scores (2*true positive/ (2*true positive + false positive + false negative)) were calculated, in addition to common model performance measures such as sensitivity, specificity, and area under the curve receiver operating curve (AUCROC) on the testing dataset. Utilization of simple accuracy or AUCROC in this imbalanced dataset would falsely favor a model that misclassified all patients as not having a VTE. The final models provided are based on the full dataset.

## Results

Of 10,926 admitted adult blunt trauma patients meeting inclusion criteria during the 8-year time period, 177 had VTE events, with a maximal incidence of 2.6% in 2012 and a minimal incidence of 1.0% in 2019. Median age (62 vs. 67 years) and frequency of two or more NTDS comorbidities were comparable in the VTE vs. no VTE groups. However, the incidence of disseminated cancer and/or cancer currently receiving chemotherapy was significantly higher in the VTE group vs. no VTE group (3.4% vs. 1.1%). The VTE group had a greater frequency of male patients (67.8% vs. 53.0%); central lines (32.8% vs. 5.1%); operating room surgical procedures (87.6% vs. 45.2%); IVC filters (35% vs. 0.15%); and non-VTE complications (45.2% vs. 8.2%) (Table 1).

**Table 1:** Demographics and Outcomes

Variable	VTE (n = 177)	No VTE (n = 10,749)	P
Age (years, median, IQR)	62 [48–79]	67 [45–82]	0.32
Male Sex	67.8%	53.0%	0.0001
Two or more comorbidities	50.3%	52.4%	0.63
Anticoagulant	10.2%	11.5%	0.65
Antiplatelet	10.7%	7.6%	0.16
Anticoagulant + Antiplatelet	18.6%	18.2%	0.97
Disseminated cancer and/or receiving chemotherapy	3.4%	1.1%	0.002
Motor vehicle crash + motorcycle crash	13.6%+22.6%	17.5%+5.5%	0.0005
Fall	49.7%	61.8%	0.003
Other	14.9%	15.1%	0.96
Body Mass Index^$^ (median, IQR)	28.4 [24.6–32.0]	26.2 [23.3–29.8]	< 0.0001
Injury Severity Score	16 [9-27]	9 [5-14]	< 0.0001
Abdomen Abbreviated Injury Score (AIS) ≥ 3	12.4%	4.5%	< 0.0001
Head/neck AIS ≥ 3	39.0%	22.3%	< 0.0001
Face AIS ≥ 3	1.1%	0.2%	0.07
Chest AIS ≥ 3	29.9%	17.0%	< 0.0001
Extremity AIS ≥ 3	50.3%	25.7%	< 0.0001
External AIS ≥ 3	0.6%	0.1%	0.18
Surgery in Operating Room (%)^$$^	87.6%	45.2%	< 0.0001
Inferior Vena Cava filter (%)	35.0%	0.15%	< 0.0001
Central line/Peripherally inserted central catheter (%)	32.8%	5.1%	< 0.0001
Mechanical Ventilation (%)	47.4%	9.9%	< 0.0001
Intensive Care Unit stay (%)	68.9%	27.0%	< 0.0001
Hosp LOS (days, median, IQR)	21 [14–36]	6 [4-10]	< 0.0001
Intensive Care Unit LOS (days, median, IQR)	14 [7-22]	0 [0–2]	< 0.0001
Non-VTE complications (%)	45.2%	8.2%	< 0.0001
Mortality (%)	6.8%	2.8%	0.004
Chemoprophylaxis (%)*			
None	41.8%	24.7%	< 0.0001
subcutaneous heparin	42.9%	54.9%	0.002
prophylactic enoxaparin	13.5%	18.4%	0.12
coumadin	0.56%	0.8%	0.52
miscellaneous	1.1%	0.6%	1
VTE Location (%)			
Infrageniculate only	13.0%	n/a	n/a
Suprageniculate only	22.6%	n/a	n/a
Infrageniculate + suprageniculate	24.9%	n/a	n/a
Lower extremity NFS	0.56%	n/a	n/a
Upper extremity	5.6%	n/a	n/a
Upper extremity + infrageniculate + suprageniculate	0.56%	n/a	n/a
PE only	24.9%	n/a	n/a
PE + infrageniculate	2.2%	n/a	n/a
PE + suprageniculate	1.1%	n/a	n/a
PE + infrageniculate + suprageniculate	3.9%	n/a	n/a
PE + upper extremity	0.56%	n/a	n/a
Days from admission to index VTE (median, IQR)	6 [3-11]	n/a	n/a
≤1 day from admission to index VTE	7.3%	n/a	n/a

^$^11 VTE and 1233 No VTE patients had no BMI available

^$$^Surgery in operating room indicates any surgical procedure other than IVC filter

*Chemoprophylaxis for VTE patients indicates: (1) agent administered at least 1 day prior to VTE event; (2) of 76 VTE patients on heparin, 13 received a combination of sq heparin followed by enoxaparin on the day prior to the VTE; (3) miscellaneous category consists of heparin drip in 2 patients. For no VTE patients, chemoprophylaxis indicates: (1) index agent utilized following admission; (2) the miscellaneous category includes 49 patients who received Factor Xa inhibitors, 49 patients who received direct thrombin inhibitors, fondaparinux, and others

**Fig. 1:**
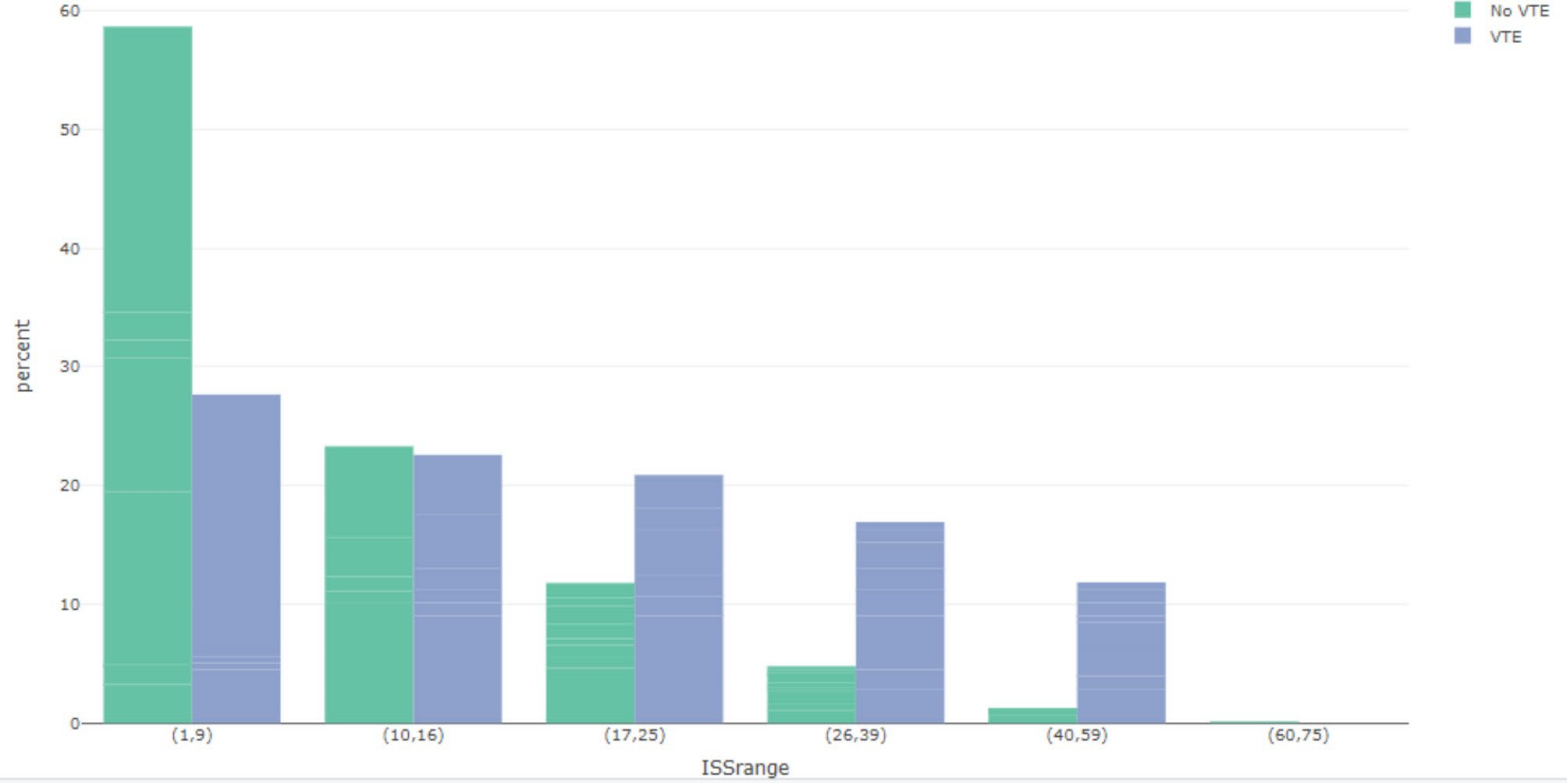
Percentage of VTE events by ISS range Green bars indicate the percentage of no VTE patients at each ISS range. Blue bars indicate percentage of VTE patients by ISS range. While proportionately more VTE events occur with increasing ISS, a substantial percentage of VTE events occur at the lower spectrum of injury severity

VTE patients had longer ICU and hospital lengths of stay (LOS). They had higher mechanical ventilation and mortality (6.8% vs. 2.8%) rates. VTE patients also had higher ISS (16 vs. 9) and concomitantly more frequently severe injuries – as indicated by AIS > = 3 – in head/neck, chest, abdomen, and extremity body regions, as compared with no VTE patients (Table 1). The most common mechanisms of injuries among our patients in both VTE and no VTE groups included falls and motor vehicle/motorcycle crashes. While the percentage of patients with VTE events generally increased in relationship to the percentage of patients at a given ISS range, VTE events occurred throughout the range of ISS, with the highest frequency in those with ISS 4–9 (n = 49, 27.7%). Specifically, 12/49 patients had ISS < = 8 and 37 patients had ISS 9. Subcategorizing VTE patients with ISS 4–9, 31/49 patients had extremity AIS > = 3 and 3/49 patients had head/neck AIS > = 3. This was followed by ISS 10–16 (n = 40, 22.6%) and ISS 17–25 (n = 37, 20.9%). Hence, 50.3% of patients who developed VTE had ISS < = 16 (Fig. 1).

The most frequently used chemoprophylaxis agent was subcutaneous heparin in both groups (VTE 42.9% vs. 54.9% in the no VTE group, p = 0.002), Table 1. This was followed by enoxaparin (VTE 13.5%, no VTE 18.4%, p = 0.12). No chemoprophylaxis was used in 41.8% of VTE patients and 24.7% of no VTE patients (p < 0.0001). A sequential combination of subcutaneous heparin and enoxaparin was used in 13 patients who subsequently developed VTE. Of the 13 patients, a reason for the change could be identified in 5 patients: one patient was felt to be at high-risk for VTE; two patients were transitioned to enoxaparin post-operatively; two patients had developed infrageniculate DVT that were not treated with therapeutic anticoagulation. For VTE patients, the median ISS for the enoxaparin group was 9 [IQR 9–14], 15.5 [IQR 9–27] for the heparin group, and ISS of 18.5 [IQR 14–30] for the no prophylaxis group, p < 0.0001.

VTE events occurred at a median of 6 days following admission, with 7.3% (n = 13) occurring within 1 day of admission, including one that occurred on the day of admission. Three of these patients had PE alone, while one had DVT with PE. With regards to VTE location, 24.9% of 177 VTE patients had PE only, and 7.8% had PE with DVT. The remainder had DVT in the following locations, in order of decreasing frequency: suprageniculate + infrageniculate; suprageniculate; infrageniculate; and upper extremity. With regards to IVC filter placement, a total of 78 IVC filters were placed, 62 in VTE patients and 16 in no VTE patients. Amongst the no VTE patients receiving IVC filters, seven had severe head/neck injuries (AIS > = 3), two of whom were discharged to spinal cord injury rehabilitation, and an additional six had severe extremity injuries.

A linear correlation matrix of risk factors for VTE demonstrated that variables most highly correlated with VTE were total number of complications excluding VTE events (r = 0.17) and mechanical ventilation (r = 0.15). Predictive multivariable classification algorithms were developed to identify risk factors for VTE. Of note, body mass index (BMI) could not be used in these analyses because approximately 11% of patients did not have this value. Logistic regression demonstrated that significant predictors with the highest adjusted odds ratios for VTE were any surgery in the operating room (odds ratio (OR) 4.98); ICU admission (OR 3.52); motor vehicle/motorcycle crash (OR 1.78); male sex (OR 1.74); and extremity AIS (OR 1.52), whereas heparin (OR 0.26) and enoxaparin (OR 0.26) were both found to be protective against VTE in the full model (Table 2). Additional significant predictors included the total number of non-VTE complications and patient age (Table 2). The AUCROC for the test dataset was 0.82. The model had a balanced accuracy of 0.80, with probabilistic odds threshold determined by the Youden index (Table 3).

**Table 2:** Multivariable logistic regression on the whole dataset

Characteristic	Adjusted Odds Ratio [Interquartile Range]	p-value
Age	1.01 [1.00, 1.02]	0.005
Male Sex	1.74 [1.22, 2.49]	0.002
Other mechanism of injury	Reference	
Fall	1.34 [0.78, 2.36]	0.30
MVC/MCC	1.78 [1.08, 3.02]	0.03
Head/neck AIS	1.02 [0.91, 1.14]	0.80
Face AIS	1.06 [0.82, 1.36]	0.60
Chest AIS	0.94 [0.83, 1.07]	0.40
Abdomen AIS	0.98 [0.84, 1.14]	0.80
Extremity AIS	1.52 [1.31, 1.77]	< 0.001
External AIS	0.99 [0.74, 1.31]	> 0.90
Number Comorbidities	0.91 [0.80, 1.02]	0.12
No chemoprophylaxis	Reference	
Heparin	0.26 [0.18, 0.38]	< 0.001
Enoxaparin	0.26 [0.15, 0.43]	< 0.001
Mechanical ventilation	1.55 [0.96, 2.53]	0.08
ICU stay	3.52 [2.21, 5.59]	< 0.001
Surgery in Operating Room	4.98 [3.08, 8.38]	< 0.001
Central Line	1.34 [0.85, 2.09]	0.20
Total Non-VTE Complications	1.46 [1.22, 1.72]	< 0.001

Only patients on prophylactic doses of heparin or enoxaparin and those not receiving chemoprophylaxis were included

**Table 3:** Performance of statistical models in predicting in-hospital VTE events, as measured on a sample testing dataset (20% of patients)

	Logistic	Random forest	GBM	Logistic Synthetic	Random Forest synthetic	GBM synthetic
TPR	0.64	0	0.48	0.64	0.45	0.27
TNR	0.83	1	0.88	0.83	0.93	0.98
FPR	0.95	0	0.94	0.90	0.05	0.88
FNR	0.01	0.02	0.01	0.01	0.08	0.01
Accuracy	0.82	0.98	0.87	0.83	0.92	0.97
Balanced Accuracy	0.80	0.50	0.68	0.74	0.69	0.62
F1 score	0.10	n/a	0.10	0.10	0.16	0.16
AUCROC	0.82	0.78	0.82	0.86	0.84	0.89

Balanced accuracy is defined as (sensitivity + specificity)/2

The F1 score is 2*True Positive/(True Positive + True Positive + False Positive + False Negative)

TPR – true positive rate (i.e. sensitivity), TNR – true negative rate (i.e. specificity), FPR – false positive rate, FNR – false negative rate

Random forest and GBM algorithms were examined to determine if model performance could be improved (Table 3). The random forest model performed poorly, as it failed to identify any patients with VTE. Meanwhile GBM had a balanced accuracy of 0.68 with a probabilistic odds threshold for classification of 0.02 based on the Youden index. The GBM model had a greater variation in model metrics than the random forest model, but the variables with the highest relative influence on accuracy were age, total non-VTE complications, extremity AIS, and central line.

Finally, the fully balanced synthetic data models were analyzed (Table 3). In logistic regression with scaled synthetic data (and hence different odds ratios), the highest odds ratios were for surgery in the operating room (OR 1.75), extremity AIS (OR 1.50), and ICU stay (OR 1.36), whereas heparin (OR 0.63) and enoxaparin (OR 0.86) had the lowest odds ratios, p < 0.001 for all of these predictors. Other significant predictors included age (OR 1.19), head AIS (OR 1.18), mechanical ventilation (OR 1.15), central line (OR 1.14), male sex (OR 1.12), fall (OR 1.10) abdomen AIS (OR 1.09), and number of comorbidities (OR 0.93). The AUCROC of the model was 0.86, with a balanced accuracy of 0.74. Performance of the random forest model with synthetic data improved, with a sensitivity of 0.45, balanced accuracy of 0.69, and F1 score of 0.16. It identified total non-VTE complications, central line, mechanical ventilation, and surgery as most important via mean decrease in accuracy. The GBM model with synthetic data had a sensitivity of 0.27 with balanced accuracy of 0.62, but this model had substantial performance variation with the scaled testing data. It identified total non-VTE complications, central line, mechanical ventilation, and surgery as the factors with highest relative influence.

## Discussion

The aim of this study was to further define blunt trauma patients at risk for VTE. Our findings augment the trauma literature in noting that: (1) While VTE developed throughout the range of ISS, the lowest ISS score group (ISS 4–9) accounted for 27.7% of VTE patients; 2) Heparin and enoxaparin were associated with reduced VTE development; and 3) Approximately 7.3% of VTE occurred within 1 day of admission.

Increased ISS scores correlated with increased risk of VTE events in agreement with existing literature, however, approximately half of our VTE patients had an ISS < = 16, including 27.7% of VTE patients with an ISS < = 9. Of note, 81.6% of all patients in this study had ISS < = 16. Current literature posits that severe injury is a risk factor for VTE, with VTE incidence increasing with injury severity. A German trauma registry study reported a 1.2% incidence rate in patients with ISS < 25; 2.1% in ISS 25–34; 2.8% in ISS 35–49; and 4.1% in ISS 50–75 [15]. The study excluded patients with ISS < 9 [15]. Another study reported a VTE incidence of 1.0% in patients with ISS < 16, 4.5% in patients with ISS > = 16; and 7.7% in patients with ISS > = 25 [16]. A retrospective analysis of trauma patients with lower extremity fractures found that major trauma patients (ISS > 15, mean ISS = 26) had an approximately six-fold increased rate (6.8% vs. 1.17%) of VTE as compared with minor trauma patients (ISS < 15, mean ISS = 4) [17]. The most common serious injury (i.e. AIS > = 3) in VTE patients with ISS < = 9 in our study was in the extremities (63.3%). Research supports the importance of extremity injuries in VTE events. In a National Trauma Database databank study, extremity AIS > = 3 had an OR of 1.96 for VTE events in a multivariable model [18]. Another study found that any extremity fracture had 2.4 OR for VTE in multivariable analyses [19].

Thus, less severely injured patients, especially those with extremity injuries, are also at elevated risk for VTE events, and any surveillance and prophylactic strategies should include these patients or will risk omitting a large proportion of the target population. This finding underlines the importance of early mobility for this group, which is likely more able to comply with increased mobility expectations. Because these patients might traditionally be considered low-risk for VTE among the broader blunt trauma population, this finding also raises challenging questions about surveillance strategies and surveillance bias through increased venous duplex ultrasound use [7, 13].

This study was not designed as a non-inferiority study. Hence, we can only conclude that both heparin and enoxaparin reduced the adjusted odds ratio for VTE development. Previous research has generally indicated that heparin chemoprophylaxis is inferior to enoxaparin chemoprophylaxis in general trauma patients [20–22]. The orthopedic trauma literature, while acknowledging practice pattern variation, also indicated limited heparin efficacy in VTE prevention [23, 24]. In contrast, a recent randomized trial in trauma patients determined that thrice daily unfractionated heparin may be non-inferior to low molecular weight heparin in VTE prevention [25]. Anti-Xa level based enoxaparin dosing has been suggested to improve enoxaparin efficacy [2, 6, 26, 27]. In contrast, a randomized study of trauma patients found no benefit to anti-Xa level-based enoxaparin dosing [28]. During the study time period, our institution infrequently used anti-Xa level based enoxaparin dosing. Rather than chemoprophylaxis agent alone, a multi-center study indicated that it may be the sum of all prophylactic measures that determine VTE incidence, where only an expectation and/or culture of mobility within institutions was associated with reduction in VTE incidence [11]. To this end, physical and occupational therapy are routinely ordered for trauma patients at our institution. Finally, the baseline VTE rate may influence the efficacy of chemoprophylaxis, where VTE incidence is dependent on duplex screening strategies, type of injury, and institutional VTE prophylaxis protocols, among others [2, 4, 8, 11, 13, 29, 30]. Our overall 1.6% VTE incidence rate was on the lower end of the broad range described in trauma literature [2–10].

It should be noted that our institution’s chemoprophylaxis dosing regimen during the study period of 2012–2019 reflects practices prior to those recently published by WTA and AAST that generally recommend 40 mg enoxaparin every 12 h, with considerations for age, creatinine clearance, and BMI, amongst others [2, 6]. Our patients received lower doses of enoxaparin, with a caveat that 52.3% of our patients were age 65 years or older and patients receiving enoxaparin had a lower ISS than those receiving heparin.

Not surprisingly, a substantial number of patients in our study who developed VTE did not receive chemoprophylaxis prior to the index event (n = 74, 41.8%). As expected, these 74 patients were severely injured (median ISS 18.5), with the head/neck being the most frequent severely injured region (n = 48) and abdomen being the least frequent severely injured region (n = 4). In contrast, in the 2,657 patients without VTE who did not receive chemoprophylaxis, the median ISS was 9 [IQR 5–16)], p < 0.0001.

Because of the rarity of the VTE events (< 2% of the admitted blunt trauma patients), we used the Youden index to increase sensitivity and balanced accuracy for VTE classification in logistic regression and GBM models. We also created a fully balanced synthetic dataset to address the class imbalance. The synthetic dataset had limited effects on the performance of logistic regression models where classification thresholds were based on Youden index. The synthetic dataset had substantial benefits for random forest classification as measured by the F1 score. Albeit balanced accuracy could be improved by the methods used to address the imbalanced dataset, the relatively low F1 scores of all models indicate difficulty in increasing both sensitivity and positive predictive value. In terms of ease of interpretation, model simplicity and general performance, the multivariable logistic regression analysis of risk factors optimized by Youden index threshold for classification is favored.

In examining additional risk factors for VTE development in logistic regression we found older age; male sex; extremity AIS; mvc/mcc mechanism of injury; surgery; number of complications; and ICU stay to be important. The gender findings contrast to a study that demonstrated no difference in VTE rates in trauma patients grouped by sex [31]. Possible reasons for these discrepant findings include differing ages and injury severities between studies. However, our results aligned with another study that noted male sex as a VTE risk factor [32]. The CLOTT study identified age, major head injury, pelvic fractures, femoral vein lines, and major venous injury as DVT risk factors [5]. The failure of logistic regression analyses to identify head/neck injury as a major risk factor and its comparatively lesser importance in other models may in part be because of some collinearity between severe head/neck injury and lack of chemoprophylaxis. Central lines were not significant predictors in logistic regression but did have high influence in random forest and GBM models with synthetic data.

Several of the above as well as other risk factors have been bundled into risk assessment scoring systems for VTE for the trauma population, such as the Greenfield Risk Assessment Profile and the Trauma Embolic Scoring System (TESS) [1, 2, 12, 33]. TESS includes 5 risk factors (obesity, ventilator duration > 3 days, lower-extremity trauma, age, and ISS); our data affirms the importance of several of these findings (age, extremity AIS, mechanical ventilation, among others) [1, 2]. AAST guidelines, however, concluded that none of these scoring systems are necessary as most injured patients requiring hospitalization for over 24 h are at increased VTE risk and therefore chemical prophylaxis should be initiated promptly [2]. The high adjusted odds ratio for surgical patients may be partly explained as an injury requiring surgery and subsequent, especially multiple, surgeries requiring anesthesia could pose a double hit on VTE pathogenesis [15]. The elevated odds ratio for ICU stay is likely related to more severe injuries, need for mechanical ventilation, age, among others. Both of these factors had high importance levels in multiple models. The association of VTE with number of non-VTE complications deserves further evaluation. Albeit rather infrequent, significantly more VTE patients had a history of disseminated cancer and/or current receipt of chemotherapy in this study.

Finally, 7.3% (n = 13) of index VTE events were present within 1 day of admission, which has important implications in terms of quality metrics, as these would likely not be preventable, especially if present on day 0. This concern was raised in the CLOTT study, which demonstrated that 1/4 of pulmonary thromboses were identified on index CT scan [5]. An older study also found that 4/63 PE events occurred within 1 day of injury [34]. Further, a single-center retrospective study noted that of 142 trauma patients with DVT, 55 were noted on duplex scans performed within 48 h of admission [35]. Hence, a corollary question is the duration of chemoprophylaxis necessary to achieve a steady state concentration to not just optimize anti-Xa levels, but also to effect VTE event reduction, as therapeutic anti-Xa levels may not necessarily reduce VTE events [28]?

### Limitations

This study has several important limitations. It is a single suburban trauma center study with a limited number of VTE patients, which may limit its generalizability to other institutions/situations. Classification of chemoprophylaxis as present among patients who received at least one dose on the day prior to VTE diagnosis may oversimplify variability and obscure delays in prophylaxis dosing or missed doses. This may affect comparisons between unfractionated heparin and enoxaparin groups. This study was not powered to detect differences between the groups.

## Conclusion

Our findings add to and challenge some of the existing trauma literature. First, 7.3% of VTE events occurred within the first day after admission, leaving limited time for chemoprophylaxis to take effect. The inclusion of VTEs that are present on index imaging scans questions quality metrics that classify all post-admission VTE as preventable. Second, while high ISS is a risk factor, a substantial number of VTE occurred in patients with low injury severity, leading to questions about optimal surveillance strategies. Third, prophylactic heparin and enoxaparin were associated with reduced VTE. However, our findings are not meant to draw conclusions on the efficacy of heparin vs. enoxaparin as the study was not designed for that purpose. These findings underscore the need for vigilance for VTE identification in blunt trauma patients throughout their hospitalization and VTE prevention.

## Data Availability

Reasonable requests for data sharing that meet with institutional IRB guidelines will be considered.
